# *In Vitro* Longitudinal Relaxivity Profile of Gd(ABE-DTTA), an Investigational Magnetic Resonance Imaging Contrast Agent

**DOI:** 10.1371/journal.pone.0149260

**Published:** 2016-02-12

**Authors:** Akos Varga-Szemes, Pal Kiss, Andras Rab, Pal Suranyi, Zsofia Lenkey, Tamas Simor, Robert G. Bryant, Gabriel A. Elgavish

**Affiliations:** 1 Department of Biochemistry and Molecular Genetics, University of Alabama at Birmingham, Birmingham, AL, United States of America; 2 Elgavish Paramagnetics Inc., Birmingham, AL, United States of America; 3 Heart Institute, Medical School, University of Pecs, Pecs, Hungary; 4 Department of Chemistry, University of Virginia, Charlottesville, VA, United States of America; Linköping University, SWEDEN

## Abstract

**Purpose:**

MRI contrast agents (CA) whose contrast enhancement remains relatively high even at the higher end of the magnetic field strength range would be desirable. The purpose of this work was to demonstrate such a desired magnetic field dependency of the longitudinal relaxivity for an experimental MRI CA, Gd(ABE-DTTA).

**Materials and Methods:**

The relaxivity of 0.5mM and 1mM Gd(ABE-DTTA) was measured by Nuclear Magnetic Relaxation Dispersion (NMRD) in the range of 0.0002 to 1T. Two MRI and five NMR instruments were used to cover the range between 1.5 to 20T. Parallel measurement of a Gd-DTPA sample was performed throughout as reference. All measurements were carried out at 37°C and pH 7.4.

**Results:**

The relaxivity values of 0.5mM and 1mM Gd(ABE-DTTA) measured at 1.5, 3, and 7T, within the presently clinically relevant magnetic field range, were 15.3, 11.8, 12.4 s^-1^mM^-1^ and 18.1, 16.7, and 13.5 s^-1^mM^-1^, respectively. The control 4 mM Gd-DTPA relaxivities at the same magnetic fields were 3.6, 3.3, and 3.0 s^-1^mM^-1^, respectively.

**Conclusions:**

The longitudinal relaxivity of Gd(ABE-DTTA) measured within the presently clinically relevant field range is three to five times higher than that of most commercially available agents. Thus, Gd(ABE-DTTA) could be a practical choice at any field strength currently used in clinical imaging including those at the higher end.

## Introduction

High field MRI scanners have gained an important role in clinical imaging in the course of the past decade [1–3]. These high field scanners, and the recently emerging ultrahigh field scanners offer improved temporal and spatial resolution, as well as an increased signal to noise ratio (SNR), as SNR positively correlates with field strength [4, 5].

Paramagnetic MRI contrast agents (CA) are used to increase contrast between tissue areas that lack an intrinsic difference in relaxation rates. A shortcoming of the presently used MRI CAs at very high-field is their steadily decreasing relaxivity with increasing magnetic field strength [6–8]. Nuclear Magnetic Resonance Dispersion (NMRD) studies have shown that gadopentetate dimeglumine (Gd-DTPA, Magnevist^®^), gadodiamide (Gd-DTPA-BMA, Omniscan^®^) and gadoversetamide (Gd-DTPA-BMEA, Optimark^®^) start showing a gradually diminishing relaxivity already at 0.1T [9, 10]. At higher, clinically relevant, fields (1.5-7T), all commercially available agents suffer from a significant loss in longitudinal relaxivity, reaching up to 80% loss of relaxivity between 1.5 to 7 T [11–13]. This negative field dependence of CA relaxivity is particularly unfortunate in light of the historic, steady increase in the typical magnetic field strength of MRI instruments in clinical use [14, 15]. Therefore, CAs with some advantage at very high magnetic fields, especially at fields above 3T, are highly desirable.

The MRI CA, Gd(ABE-DTTA), has been extensively studied for various MRI applications. The MRI applicability of Gd(ABE-DTTA) has been investigated in several canine and porcine models of acute ischemia/reperfusion and myocardial infarction [16–18]. It has been also shown that this agent has special affinity for acute infarcts, and thus it can discriminate between acute and subacute/chronic infarcts [19]. The feasibility of Gd(ABE-DTTA) enhanced MRI has also been investigated in the mouse model of early prostate cancer [20]. It has shown no deleterious physiological effects at the level of the MRI-effective dose in the canine model [21].

The purpose of the present study was the in vitro characterization of the magnetic field dependence of the longitudinal relaxivity of this CA. As a control, the longitudinal relaxivity of Gd-DTPA, a clinically approved CA, was parallelly measured.

## Materials and Methods

### Preparation of Gd(ABE-DTTA) and Gd-DTPA samples

Gd(ABE-DTTA) was synthesized as described by Saab-Ismail et al [16]. Solutions of 1:1 Gd:ABE-DTTA and Ca:ABE-DTTA were mixed in a ratio of 1:0.3 to obtain a 10 mM stock. This Gd:Ca ratio is used in live animal studies to ensure sufficient ligand availability for the CA complex without having free ligand present at any time. From the 10mM stock, 0.5mM and 1mM solutions were prepared, as the NMRD instrument requires the sample to have an expected longitudinal relaxation rate (R_1_) between 10 and 100s^-1^. The pH of the solutions was set to 7.4.

Manufacturer’s 0.5M Gd-DTPA product (Magnevist, Bayer Healthcare Pharmaceuticals Inc., Wayne, NJ) was used to prepare a 4mM solution. Based on our previous relaxivity studies, the longitudinal relaxivity of Gd(ABE-DTTA) is about four-fold higher than that of Gd-DTPA, thus the selection of a 4mM sample concentration ensured the relaxation rate being within the desired range.

For further quality assurance, the Gd concentration of the CAs was determined by inductively coupled plasma mass spectrometry (ICP-MS) independently (Galbraith Laboratories Inc., Knoxville, TN), and these concentrations were used for relaxivity calculations.

### NMRD experiments

T_1_ was measured using a Stelar SpinMaster (Mede, Italy) field cycling NMR spectrometer in the magnetic field range of 0.0002–1.0T. Samples were contained in 10 mm glass tubes, and temperature (37°C) was regulated by gas flow. The detection field was 15.8 MHz where a single 90-degree pulse was applied and the free induction decay was detected. A value was measured following each of 32 relaxation delays at each magnetic field [22].

### MRI and NMR experiments

The magnetic field dependence of the longitudinal relaxivity of Gd(ABE-DTTA) was determined between 1.5 and 20T using static field MRI and NMR instruments (Table 1). All measurements were carried out at 37°C, and were repeated four times.

**Table 1:** List of the MRI and NMR equipment used in our studies.

MRI / NMR System	Field (T)
GE Signa CV/i^a^	1.5
Philips Achieva^b^	3
Bruker ARX 300 MHz^c^	7
Bruker DRX 400 MHz^c^	9.4
Bruker Avance III 600MHz^c^	14
Bruker Avance II 700 MHz^c^	16.4
Bruker Avance III 850 MHz^c^	20

^a^GE Healthcare, Milwaukee, WI

^b^Philips Healthcare, Best, The Netherlands

^**c**^Bruker Corporation, Billerica, MA

For the MRI studies, samples were created using 50 mL polypropylene centrifuge tubes filled with 30 mL CA, and placed in a glass beaker. Polystyrene foam was used to secure the tubes in the beaker, and the interior of the beaker was filled up with tap water. The beaker was placed in a 37°C water bath, and its temperature was maintained by preheated (37°C) hot packs during the MRI session. Inversion recovery (IR) gradient echo sequences were used to measure T_1_ using the following parameters: field of view 160mm, image matrix 256^2^, slice thickness 7mm, flip angle 25°, and echo and repetition time 3.19/7.19ms and 2.04/3.34ms, at 1.5 and 3T, respectively. On the 1.5T instrument, 24 inversion times (TI) between the range of 50 and 1100ms were used, while on the 3T system, 24 TIs between 15-1300ms were applied.

For the NMR studies, samples were placed in 1.5 mm O.D. tubes inserted coaxially within 5 mm O.D. NMR tubes which contained deuterated water (D_2_O) for field locking purposes. In the NMR instruments, N_2_ flow and a heater copper coil were used for temperature control. Temperature was calibrated by methanol ^1^H chemical shift measurements [23]. NMR measurements were performed using 17 delay times between 1 and 3000ms.

### Relaxation rate calculation

MRI data sets were analyzed by using dedicated cardiac MRI software (MASS Research Software, Leiden University Medical Center, The Netherlands). T_1_ was measured based on regions of interest placed in the middle of each tube, and the built-in T_1_ fitting function was implemented. NMR data were analyzed by the TopSpin software (Bruker Corporation, Billerica, MA) available on the NMR consoles.

T_1_ values were calculated from the SI vs. TI dependence by an automated procedure, applying a non-linear, three-parameter least-squares curve-fitting routine as described previously [24]. The relaxivity was expressed based on the calculated R_1_ and the concentration of the CA.

## Results

The true Gd concentration of the nominal 0.5mM and 1mM Gd(ABE-DTTA) and 4mM Gd-DTPA solutions determined by ICP-MS were 82, 154 and 657ppm, respectively, from which the actual sample concentrations of 0.52mM, 0.97mM and 4.17mM, respectively, were calculated. These values were in turn used for the relaxivity calculations.

**Fig 1:**
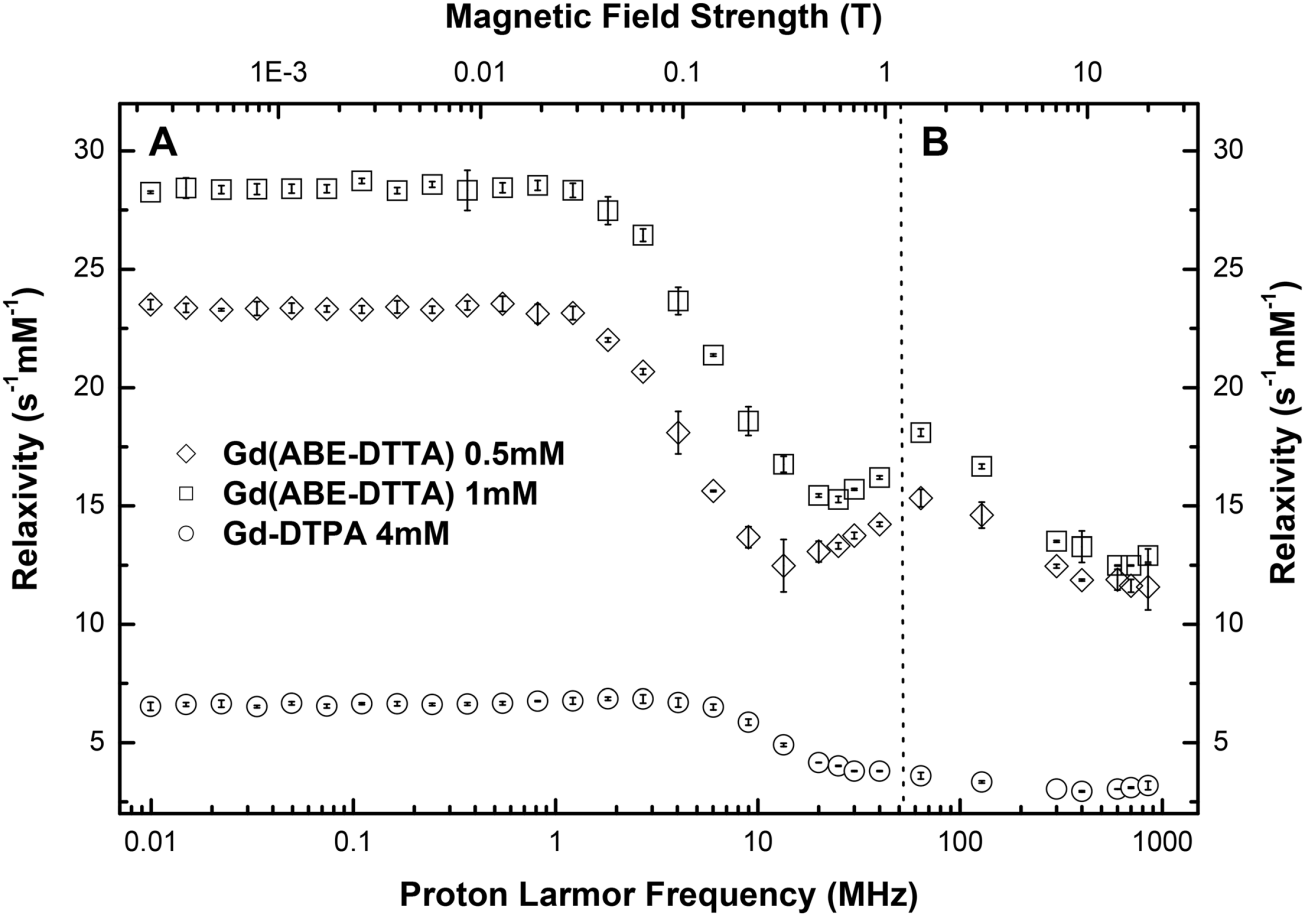
Longitudinal relaxivity profile of Gd(ABE-DTTA) and Gd-DTPA. **A:** The field dependency of the longitudinal relaxivity of 4 mM Gd-DTPA (circles) and 0.5 mM and 1 mM Gd(ABE-DTTA) (diamonds and squares, respectively) acquired with NMRD is shown. **B:** The relaxivity of Gd(ABE-DTTA) and GD-DTPA obtained on a series of static field MRI and NMR instruments listed in Table 1, as a function of magnetic field strength is shown. All measurements were carried out at 37°C and pH 7.4.

The relaxivity obtained from the NMRD data, and data acquired by seven different single-field instruments are compiled in Table 2 and also plotted as a function of magnetic field strength in Fig 1. Similarly to other CAs, Gd(ABE-DTTA) shows its maximum relaxivity (~23 s^-1^mM^-1^ and ~ 28s^-1^mM^-1^ at a concentration of 0.5 and 1mM, respectively) at magnetic field strengths less than 0.01T. Also similarly to other CAs, its relaxivity starts decreasing at about 0.015T, but contrary to most clinically used CAs, increases again reaching a local maximum of 18.1 s^-1^mM^-1^ at 1.5T, coinciding with the magnetic field still used most widely in clinical practice. This value is considerably larger than that of most other CAs. Similarly to typical CAs presently used in the clinic, its relaxivity above this point again decreases with field strength. Nevertheless, even at 7T its relaxivity is still at the considerably high level of ~13s^-1^mM^-1^ using both concentration levels tested by us, and showing a minimal decrease when reaching 20T (~12s^-1^mM^-1^), the highest field employed in the present study.

**Table 2:** Longitudinal relaxivity data (s^-1^mM^-1^) of Gd(ABE-DTTA) and Gd-DTPA obtained on NMRD, MRI and NMR instruments are shown along with the corresponding field strength (Tesla) and proton Larmor frequency (MHz).

			Relaxivity (s^-1^mM^-1^)		
Equipment	Field (T)	Frequency (MHz)	Gd(ABE-DTTA) 0.5mM	Gd(ABE-DTTA) 1mM	Gd-DTPA 4mM
**NMRD**	0.0002	0.0099	23.51±0.21	28.25±0.05	6.54±0.17
	0.0003	0.014	23.37±0.19	28.43±0.43	6.62±0.08
	0.0005	0.022	23.29±0.05	28.36±0.17	6.65±0.15
	0.0007	0.033	23.34±0.29	28.38±0.23	6.53±0.05
	0.0011	0.049	23.36±0.21	28.40±0.19	6.67±0.07
	0.0017	0.073	23.32±0.13	28.40±0.17	6.55±0.08
	0.0025	0.11	23.30±0.16	28.73±0.11	6.65±0.04
	0.0038	0.16	23.41±0.26	28.32±0.13	6.65±0.09
	0.0057	0.24	23.29±0.15	28.58±0.12	6.62±0.05
	0.0086	0.36	23.47±0.19	28.33±0.85	6.64±0.06
	0.012	0.54	23.54±0.32	28.44±0.21	6.67±0.07
	0.019	0.81	23.12±0.40	28.54±0.21	6.76±0.02
	0.028	1.21	23.15±0.28	28.33±0.30	6.77±0.14
	0.042	1.81	22.01±0.09	27.47±0.59	6.86±0.07
	0.063	2.7	20.67±0.12	26.44±0.26	6.85±0.18
	0.094	4	18.10±0.90	23.66±0.58	6.70±0.19
	0.14	6	15.63±0.03	21.37±0.05	6.51±0.12
	0.21	8.9	13.68±0.44	18.59±0.61	5.87±0.13
	0.31	13.4	12.48±1.11	16.76±0.34	4.91±0.07
	0.46	20	13.08±0.44	15.44±0.07	4.16±0.01
	0.58	25	13.32±0.13	15.27±0.13	4.02±0.02
	0.7	29.9	13.75±0.13	15.70±0.04	3.80±0.02
	0.93	40	14.22±0.09	16.20±0.07	3.80±0.01
**MRI**	1.5	64	15.33±0.37	18.10±0.16	3.60±0.14
	3	128	11.87±0.04	16.67±0.10	3.34±0.05
**NMR**	7	300	12.46±0.08	13.51±0.04	3.04±0.00
	9.4	400	11.87±0.04	13.28±0.66	2.94±0.03
	14	600	11.89±0.02	12.49±0.02	3.04±0.01
	16.4	700	11.63±0.27	12.48±0.02	3.10±0.03
	20	850	11.58±0.97	12.91±0.28	3.19±0.18

The longitudinal relaxivity of Gd-DTPA, parallelly measured at the same magnetic fields as Gd(ABE-DTTA), are shown in Table 2 and Fig 1. The highest relaxivity (6.8s^-1^mM^-1^) was observed at the very low field range, while the relaxivity at the clinically relevant field strengths dropped below 3.6s^-1^mM^-1^.

## Discussion

The relaxivity of the clinically available CAs, including Gd-DTPA, the most often used MRI CA, decreases with increasing field strength [11–13]. The magnitude of the loss in relaxivity varies among the CAs between 10 and 80%, with gadobutrol (11.5% decrease from 1.5T (5.2 s^-1^mM^-1^) to 7T (4.6 s^-1^mM^-1^)) at the lower, and gadofosveset (79.1% decrease from 1.5T (27.7 s^-1^mM^-1^) to 7T (5.8 s^-1^mM^-1^)) at the higher end of the range [11, 12]. Gd-DTPA is located in the lower end of this range, as it shows a ~15.3% drop in relaxivity when measured at 7T (3.3 s^-1^mM^-1^). vs. at 1.5 T (3.9 s^-1^mM^-1^).

Gd(ABE-DTTA) demonstrates a local maximum in relaxivity at a relatively high field strength, around 1.5T, then gradually decreases at higher magnetic fields (Table 2, Fig 1). The decrease in relaxivity in the clinically relevant field range (between 1.5 and 7T) is ~25% (from 18.1 s^-1^mM^-1^ at 1.5T to 13.5 s^-1^mM^-1^ at 7T). The high relaxivity of Gd(ABE-DTTA) at the lower end of this field range (1.5T) is seconded only by the relaxivity of gadomer (17.3 s^-1^mM^-1^) but is three to five times higher than the relaxivity of all other CAs [12, 13]. At the higher end of the clinically relevant field range (7T), Gd(ABE-DTTA)’s relaxivity is two to four times higher than those of all available CAs, including gadomer. While the longitudinal relaxivity of gadofosveset in plasma reaches a similar level at 1.5T, the relaxivity of this CA significantly drops at even higher fields [13]. For quality control purposes, and to highlight the unique field dependency of the Gd(ABE-DTTA) relaxivity, we compared the relaxivity profile of Gd(ABE-DTTA) to that of Gd-DTPA (Table 2, Fig 1). The relaxivity profile of Gd-DTPA measured at 37°C was comparable to the relaxivity values reported in the literature [13, 25]. NMRD measurements of Gd-DTPA in water at 35°C in the field range below 1T, reported by Kellar et al., showed good agreement with our mesurements at 37°C [25]. Relaxivity data at higher fields (1.5 and 3T) published by Rohrer et al. also demonstrated similar values to ours [13]. Very limited data are available from ultra-high magnetic fields for comparison with our results. Noebauer-Huhmann et al. reported the longitudinal relaxivity of Gd-DTPA (7T, 37°C) diluted in plasma [12]. The difference in the measured relaxivity in distilled water (3.0 s^-1^mM^-1^) and the reported relaxivity in plasma (3.3 s^-1^mM^-1^ [12]) arises from the fact that the T_1_ relaxivity of the majority of Gd CAs in plasma is generally higher than in distilled water [13].

The theory of the field dependence of the relaxivity of paramagnetic ions and their complexes, including Gd^3+^ chelates, is well established and is based on the Solomon-Bloembergen-Morgan (SBM) equations (cf. e.g., in the appendix of [26]). It is governed by numerous factors having complex relationships with each other, namely the inner coordination environment of the Gd^3+^ ions and the number of water molecules in the Gd^3+^ inner coordination sphere; the field dependent relaxation time of the gadolinium unpaired electron spins (τ_S_), the rotational correlation time (τ_R_) that describes the rotational reorientation of the gadolinium complex, (τ_V_), a parameter that characterizes the fluctuations that govern the electron spin relaxation rate and determines the dependence of τ_S_ on the static magnetic field; the average distance between the Gd^3+^ and the water protons in the inner sphere; the residence time (τ_M_) of the coordinated water molecules; and the diffusional correlation time (τ_D_) of a solvent water molecule in the outer sphere [9]. The role of the second hydration shell can be important only when the ligand exchange in the inner sphere is slow [26]. The field dependence profile of Gd(ABE-DTTA) below 0.4T looks qualitatively similar to that of Gd-DTPA, and above this field it shows features similar to those of protein-bound paramagnetic ions, for example Gd-concavalin A, Mn-concavalin A and Cu-SOD in water solutions, reported by Koenig et al [26, 27]. The initial, low field inflection (that reflects a correlation time dominated by rotational reorientation) when the product of the correlation time and the electron Larmor frequency is of the order of unity, but at higher frequencies the electron relaxation time increases because of its own magnetic field dependence, modulated by τ_V_, and causes the observed local maximum in the proton relaxation rate at approx. 1.5T. Above this high magnetic field, the proton relaxation rate disperses because the product of τ_S_, the electron spin-lattice relaxation time, and the nuclear Larmor frequency approaches unity. This occurs as the product of the electron correlation time τ_V_ and the electron Larmor frequency becomes approximately unity.

Because the field dependency profile of the Gd(ABE-DTTA) relaxivity looks similar to the above mentioned high molecular weight complexes, one would expect the environment of its Gd^3+^ ion to be similar to those of such slowly-rotating complexes. As Gd(ABE-DTTA) is of the relatively low molecular weight of 763 Dalton, its relaxivity profile is at first perplexing. We suggest, however, that the chemical structure of the ABE-DTTA ligand, with its lipophilic butyryl chain, may provide the explanation. Such lipophilic chains may lead to micelle formation, or alternatively some other intermolecular complexes may form, increasing the effective radius of the aggregate complex, and leading to the observed field dependency profile which is predicated on a large τ_R_ and thus the dominance of τ_S_ in the effective correlation time. The increase in relaxivity when Gd(ABE-DTTA) concentration is increased from 0.5 to 1 mM indicates that such a mechanism is a possibility as an increase in concentration would shift the chemical equilibrium towards a higher fraction of the putative aggregate complex resulting in an increase in the observed relaxivity.

Using increasingly higher magnetic field strengths in clinical MRI instruments has been a trend from the inception of this modality. This trend has recently reached 7T, which presently seems to be useful especially for musculoskeletal, neurological, and cardiovascular applications [5, 28–34].

A CA with sufficient relaxivity at ultrahigh fields may produce needed contrast between tissue areas where one is not otherwise available. Irrespective of the exact mechanism of its relaxivity, considering the historic trend of the development of clinical MRI instruments that operate at increasingly higher magnetic fields [15], Gd(ABE-DTTA) could become the CA of choice in cases where imaging at ultrahigh fields would be particularly useful. As our results demonstrate, it would be practical to use this agent at any field strength between 1.5 and 20T.

### Limitations

In vitro relaxivity measurements were carried out in distilled water. Thus, our results do not necessarily represent the behavior of the CA in other solvents or in live tissues.

## Supporting Information

S1 Fig The molecular scheme of the ABE-DTTA ligand. (TIF)
