# Supplementary figures and images for: In Vitro Longitudinal Relaxivity Profile of Gd(ABE-DTTA), an Investigational Magnetic Resonance Imaging Contrast Agent
Source: PLoS One. 2016 Feb 12;11(2):e0149260. doi: 10.1371/journal.pone.0149260 (PMC4752229; doi:10.1371/journal.pone.0149260)

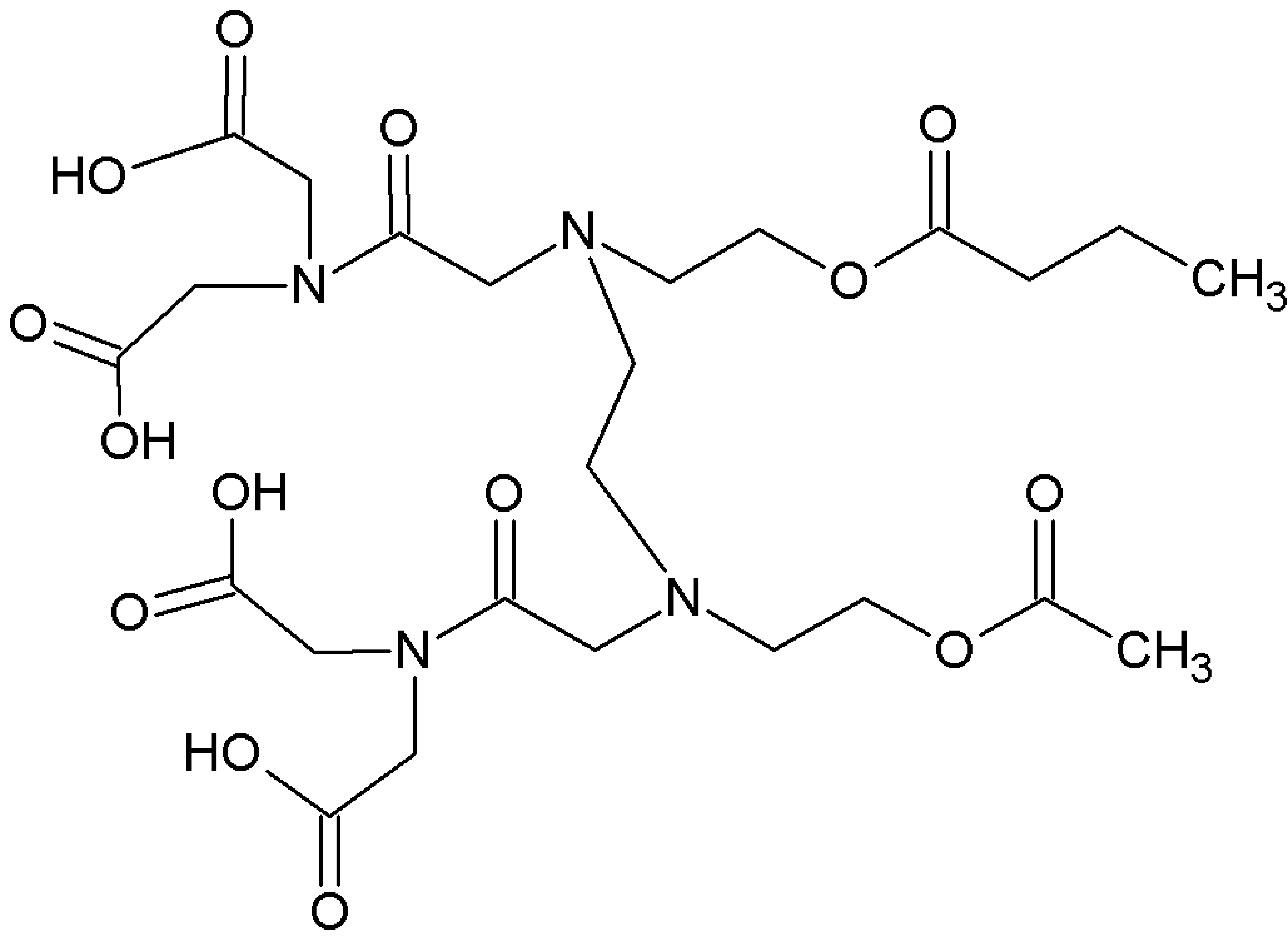

Supplement: S1 Fig — (TIF) [file pone.0149260.s001.tif]
